# The impact of influenza A(H1N1)pdm09 compared with seasonal influenza on intensive care admissions in New South Wales, Australia, 2007 to 2010: a time series analysis

**DOI:** 10.1186/1471-2458-12-869

**Published:** 2012-10-12

**Authors:** Andrea Schaffer, David Muscatello, Michelle Cretikos, Robin Gilmour, Sean Tobin, James Ward

**Affiliations:** 1 Centre for Epidemiology and Research, NSW Ministry of Health, North Sydney, NSW, Australia; 2 Centre for Health Protection, NSW Ministry of Health, North Sydney, NSW, Australia; 3 Aboriginal and Torres Strait Islander Health Program, Kirby Institute, Sydney, NSW, Australia

**Keywords:** Intensive care, Influenza, Respiratory illness, Hospitalisations, H1N1, Australia, Pandemic, Demand, Indigenous, Pregnancy

## Abstract

**Background:**

In Australia, the 2009 epidemic of influenza A(H1N1)pdm09 resulted in increased admissions to intensive care. The annual contribution of influenza to use of intensive care is difficult to estimate, as many people with influenza present without a classic influenza syndrome and laboratory testing may not be performed. We used a population-based approach to estimate and compare the impact of recent epidemics of seasonal and pandemic influenza.

**Methods:**

For 2007 to 2010, time series describing health outcomes in various population groups were prepared from a database of all intensive care unit (ICU) admissions in the state of New South Wales, Australia. The Serfling approach, a time series method, was used to estimate seasonal patterns in health outcomes in the absence of influenza epidemics. The contribution of influenza was estimated by subtracting expected seasonal use from observed use during each epidemic period.

**Results:**

The estimated excess rate of influenza-associated respiratory ICU admissions per 100,000 inhabitants was more than three times higher in 2007 (2.6/100,000, 95% CI 2.0 to 3.1) than the pandemic year, 2009 (0.76/100,000, 95% CI 0.04 to 1.48). In 2009, the highest excess respiratory ICU admission rate was in 17 to 64 year olds (2.9/100,000, 95% CI 2.2 to 3.6), while in 2007, the highest excess rate was in those aged 65 years or older (9.5/100,000, 95% CI 6.2 to 12.8). In 2009, the excess rate was 17/100,000 (95% CI 14 to 20) in Aboriginal people and 14/100,000 (95% CI 13 to 16) in pregnant women.

**Conclusion:**

While influenza was diagnosed more frequently and peak use of intensive care was higher during the epidemic of pandemic influenza in 2009, overall excess admissions to intensive care for respiratory illness was much greater during the influenza season in 2007. Thus, the impact of seasonal influenza on intensive care use may have previously been under-recognised. In 2009, high ICU use among young to middle aged adults was offset by relatively low use among older adults, and Aboriginal people and pregnant women were substantially over-represented in ICUs. Greater emphasis on prevention of serious illness in Aboriginal people and pregnant women should be a priority in pandemic planning.

## Background

In Australia in 2009, an outstanding feature of the arrival of pandemic influenza caused by the A(H1N1)pdm09 virus was an apparently strong need for intensive care services among patients infected with the virus [1]. A large number of reports have described the characteristics of intensive care patients with pandemic influenza both in Australasia [1-3] and internationally [4-6], but there has been little examination of the relative impact of the pandemic and seasonal influenza strains on use of intensive care.

In the state of New South Wales (NSW), Australia (population 7 million) the 2009 epidemic associated with A(H1N1)pdm09 influenza subtype occurred during the usual winter influenza season, and had a single distinct peak [3]. Of 225 people admitted to intensive care in NSW with confirmed infection, 87% were aged under 65 years, and included 16 pregnant women and 14 Aboriginal people [3]. Overseas experiences were similar [7,8]. In 2009, there was also a high level of severe illness, as evidenced by the development of acute respiratory distress syndrome (ARDS), and the need for extracorporeal membrane oxygenation (ECMO) [9].

Most studies have focused on laboratory confirmed or suspected influenza A(H1N1)pdm09 infection, while no studies have attempted to estimate the overall contribution of influenza to use of intensive care over time. Patients with influenza who require hospitalisation tend not to present with classic influenza-like illness (ILI), therefore relying on ILI presentations to identify patients suffering serious influenza infections or influenza-related complications is inadequate [10]. A substantial proportion of patients admitted to intensive care with pneumonia or respiratory infection are found to be positive for influenza, if an appropriate test is performed in a timely manner [11]. Broader definitions of influenza-associated illness are therefore required to estimate the full impact of influenza-related illness on health care utilisation [12,13].

For the years 2007 to 2010, we used a population database of all hospital admissions in NSW, to estimate and compare the impact of recent seasonal and pandemic influenza epidemics on admissions to intensive care in the general population, in pregnant women, and in Aboriginal people.

## Methods

### Data

The NSW Admitted Patients Data Collection (APDC) consists of routinely collected data on all hospital separations (discharges, transfers and deaths) from all NSW public and private sector hospitals and day procedure centres. Data were obtained from the Health Outcomes and Information Statistical Toolkit (HOIST), Centre for Epidemiology and Evidence, NSW Ministry of Health. The data include patient demographics, service referred to on separation, up to 50 primary and co-morbid diagnoses and up to 50 procedures applied during the admission episode. The diagnoses are recorded using the Australian modification of the International Statistical Classification of Diseases and Related Problems, 10^th^ revision (ICD 10-AM). Procedures are recorded using the Australian Classification of Health Interventions (ACHI). All admissions in the APDC with an unplanned admission to an intensive care unit (ICU) during 2007 through 2010 inclusive were extracted for analysis. No identifiable information was used for this study and so ethics approval was not required.

### Outcomes

Using diagnoses recorded for each admission, we considered three outcomes to assess the impact of influenza on intensive care admissions: influenza alone (ICD-10-AM J09 to J11), influenza or pneumonia (ICD-10-AM J09 to J18), and any respiratory illness (ICD-10-AM J00 to J99). To identify influenza and pneumonia, we used all primary and additional diagnoses. The all respiratory category included all influenza and pneumonia diagnoses, as well as a primary diagnosis of any other respiratory illness. Pregnant/postpartum women were identified by the ICD-10 codes O09 to O99, and Z33 to Z37 in any primary or additional diagnosis. In the APDC, Indigenous status is recorded based on self-assessed identification as either Aboriginal and/or Torres Strait Islander at time of admission. Greater than 95% of the NSW Indigenous population identify as Aboriginal.

To further assess the impact on intensive care, we determined the number of admissions with markers of severe illness, including respiratory failure, acute respiratory distress syndrome (ARDS), mechanical ventilation and extracorporeal membrane oxygenation (ECMO). Respiratory failure (ICD-10-AM J96) and ARDS (ICD-10-AM J80) were identified using all diagnoses. Mechanical ventilation and ECMO were identified using ACHI codes 13822–00, 13882–01, 13882–02, 13857–00 and 13879–00 for mechanical ventilation and 90225–00 for ECMO. The median hours spent in intensive care and the proportion of patients who died during their ICU stay were also determined, using information recorded in the hospitalisation data.

### Data analysis

Analysis was restricted to NSW residents only. Weekly counts of intensive care admissions for all outcomes were calculated for the study period, and weekly rates were then calculated using estimated resident populations linearly interpolated from mid-year estimates [14] and from year end projections for Aboriginal people [15]. To estimate the population of pregnant/postpartum women at risk during each influenza period, first the number of women who were pregnant in a year was calculated by multiplying the total number of confinements in that year [16] by 40/52, as an average pregnancy lasts 40 weeks. Second, to determine the number of women at risk during a given week in that year, this number was divided by 52. The resulting weekly rates were summed over the length of the influenza season to give estimated rates over the entire season.

Each influenza period was defined as the period when the number of respiratory specimens that tested positive for influenza type A and B combined exceeded 3%, which is the lowest value that gave a distinct, single epidemic period in each year. Excess intensive care admissions were estimated using a Serfling-type approach [17] to account for the baseline or underlying seasonality that occurs even when influenza epidemics are absent. A regression model was fit using robust regression (SAS ROBUSTREG), which limits the influence of outliers caused by influenza epidemics and other short term changes in mortality and thus does not require removal of the influenza periods to estimate the baseline, which is the traditional approach. This method has been used previously to estimate excess mortality due to influenza epidemics [18,19]. The traditional approach was not used as it is sensitive to the time period chosen to be excluded for estimation of the baseline. The two methods have been shown to give similar results, with the robust regression approach giving more conservative estimates [18].

The model was specified as

$$\begin{array}{c} \text{Expected}\left(Y\right)=\beta_{0}+\beta_{1}\left[t\right]+\beta_{2}\left[t^{2}\right]+\beta_{3}\left[t^{3}\right]\\ \;+\beta_{4}\left[\sin\left(\frac{2\pi t}{52.2}\right)\right]+\beta_{5}\left[\cos\left(\frac{2\pi t}{52.2}\right)\right] \end{array}$$

where *Y* represents the weekly rate of intensive care admission and *t* is the week number. *t*, *t*^2^ and *t*^3^ were included to account for linear and non-linear trends, while the sine and cosine terms account for the amplitude and phase of the annual seasonal wave.

To estimate the overall contribution of influenza-related illness to the rates of intensive care admission during each influenza epidemic period, the difference between the observed and baseline predicted admission rates in each week were calculated and then summed over all weeks of the influenza period. The 95% confidence limits for the rate differences were also calculated in a similar fashion by using the upper and lower confidence limit of each weekly estimate of the baseline as the reference from which to calculate the difference between the observed admission rates and the lower and upper confidence limits of the predicted admission rates. Excess admission counts and their 95% confidence limits were estimated by applying the rate difference to the population estimates for that year. A negative difference indicates that there were fewer admissions than expected, while a positive difference indicates that there were more admissions than expected, when compared to the seasonal baseline.

For outcomes or procedures that were rare outside of the influenza epidemic periods, or which did not have a distinct seasonal background pattern, we assumed a constant rate during the non-influenza period, which was estimated by calculating the average weekly rate over the study period excluding the influenza epidemic periods.

The fit of the model for estimating the baseline was assessed by checking the robust residuals outside of the influenza seasons. Autocorrelation plots of the residuals were also constructed. All analyses were performed using SAS V9.2.

## Results

Between 1 January 2007 and 31 December 2010 in NSW, there were 75,154 unplanned ICU admissions in NSW, or an average of 18,789 (266/100,000 population) per year.

**Figure 1:**
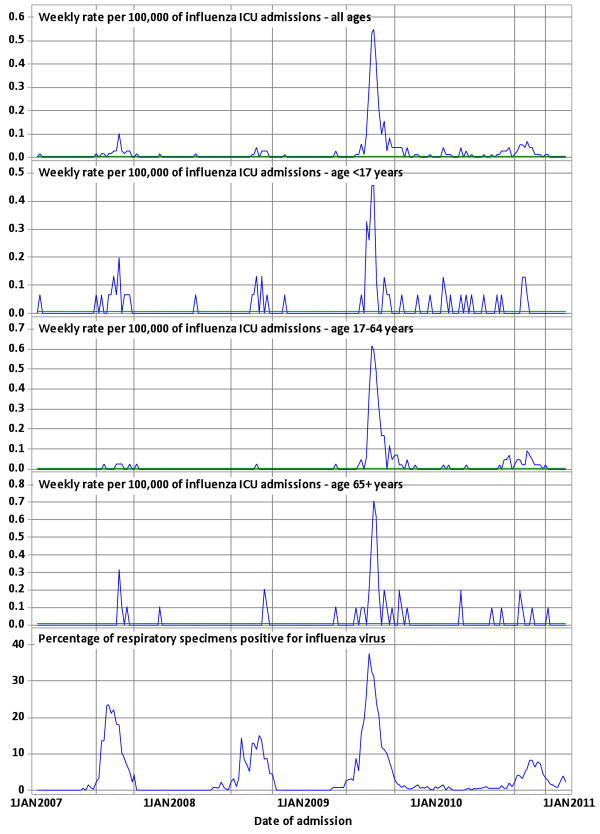
**Rates of intensive care admission with a diagnosis of influenza.** Weekly observed (blue) and predicted (green) rates of intensive care admission with a diagnosis of influenza as a primary or other diagnosis per 100,000 people, and the weekly percentage of respiratory specimens testing positive for the influenza virus, New South Wales, Australia, 2007 to 2010. Each influenza period is marked with vertical reference lines.

**Figure 2:**
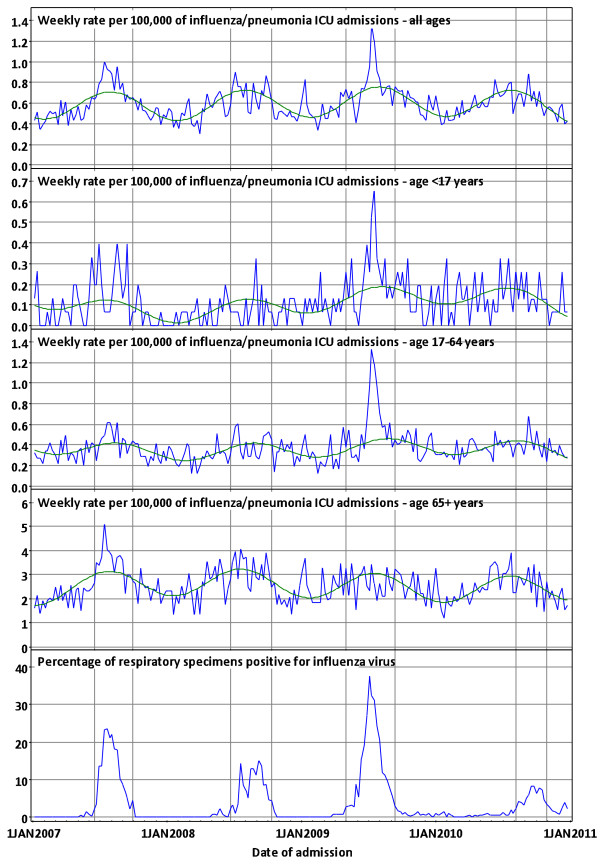
**Rates of intensive care admission with a diagnosis of influenza/pneumonia.** Weekly observed (blue) and predicted (green) rates of intensive care admission with a diagnosis of influenza or pneumonia as a primary or other diagnosis per 100,000 people, and the weekly percentage of respiratory specimens testing positive for the influenza virus, New South Wales, Australia, 2007 to 2010. Each influenza period is marked with vertical reference lines.

**Figure 3:**
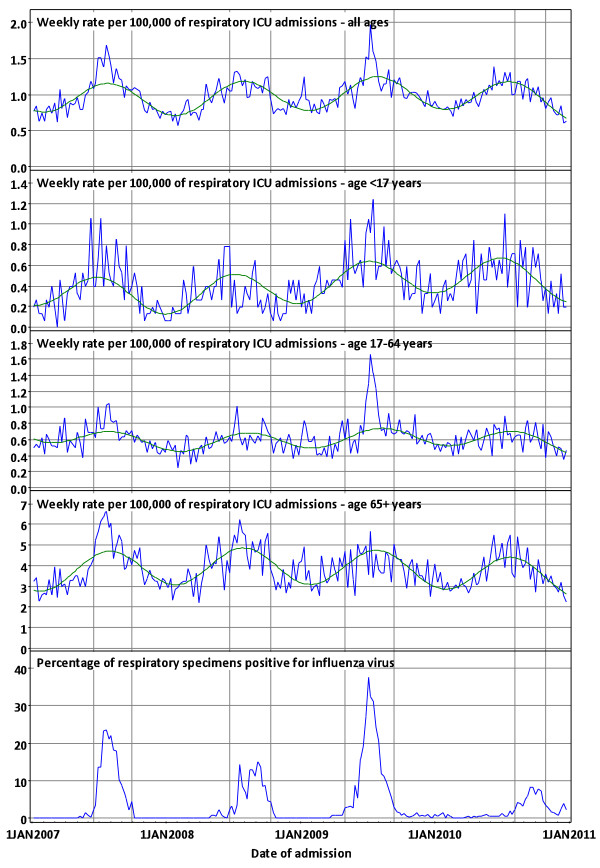
**Rates of intensive care admission with a diagnosis of any respiratory illness.** Weekly observed (blue) and predicted (green) rates of intensive care admission with a primary respiratory diagnosis or a diagnosis of influenza or pneumonia as a primary or other diagnosis per 100,000 people, and the weekly percentage of respiratory specimens testing positive for the influenza virus, New South Wales, Australia, 2007 to 2010. Each influenza period is marked with vertical reference lines.

The time series of the observed weekly rate of influenza-related intensive care admission by age group are shown in Figure 1. The observed and predicted seasonal baseline weekly rate of influenza and pneumonia-related intensive care admission and all unplanned respiratory intensive care admissions are presented by age group in Figures 2 and 3. Overall, the 2008 and 2010 influenza periods had little obvious impact on admissions to intensive care, compared with 2007 and 2009.

Compared with other years, influenza was much more commonly included as part of an intensive care admission diagnosis in 2009 (Table 1, Figure 1). In the 2009 pandemic period, there were 182 (95% CI 180 to 185) excess, unplanned ICU admissions that received a diagnosis of influenza, compared with a maximum of 28 in other epidemic periods, which was observed in 2010. By contrast, when using the broader influenza/pneumonia and all respiratory diagnosis categories, the estimated impact of influenza on intensive care admissions for all ages was greater in 2007 than in 2009 (Table 1).

**Table 1:** Estimated differences in observed and predicted rates and counts of intensive care admissions, 2007–2010

		**Influenza season**							
		**2007 (15 weeks)**		**2008 (16 weeks)**		**2009 (19 weeks)**		**2010 (12 weeks)**	
		**Rate difference per 100,000 (95% CI)**	**Count difference (95% CI)**	**Rate difference per 100,000 (95% CI)**	**Count difference (95% CI)**	**Rate difference per 100,000 (95% CI)**	**Count difference (95% CI)**	**Rate difference per 100,000 (95% CI)**	**Count difference (95% CI)**
**All ages**	Influenza only*	0.25 (0.22 to 0.27)	17 (15–19)	0.08 (0.05 to 0.11)	6 (4 to 8)	2.56 (2.52 to 2.59)	182 (180 to 185)	0.33 (0.31 to 0.35)	24 (22 to 25)
	**Influenza/pneumonia**	1.45 (0.98 to 1.91)	100 (68 to 132)	0.045 (−0.41 to 0.50)	3 (−29 to 35)	0.98 (0.41 -1.55)	70 (30 to 111)	−0.05 (−0.48 to 0.38)	−4 (−35 to 28)
	**All respiratory**	2.55 (1.96 to 3.13)	176 (135 to 217)	−0.14 (−0.71 to 0.43)	−10 (−50 to 30)	0.76 (0.043 to 1.48)	54 (3 to 105)	−0.039 (−0.58 to 0.50)	−3 (−42 to 36)
**<17 years**	Influenza only*	0.68 (0.62-0.73)	10 (9 to 11)	0.34 (0.28 to 0.40)	5 (4 to 6)	1.81 (1.75 to 1.88)	28 (27 to 29)	0.23 (0.19 to 0.28)	4 (3 to 4)
	**Influenza/pneumonia**	1.12 (0.69 to 1.54)	17 (11 to 23)	−0.62 (−1.03 to −0.21)	−9 (−16 to −3)	1.27 (0.76 to 1.79)	20 (12 to 27)	0.08 (−0.32 to 0.47)	1 (−5 to 7)
	**All respiratory**	2.11 (1.24 to 2.98)	32 (19 to 45)	−2.69 (−3.53 to −1.84)	−41 (−54 to −28)	2.01 (0.96 to 3.07)	31 (15 to 47)	0.26 (−0.54 to 1.06)	4 (−8 to 16)
**17 to 64 years**	Influenza only*	0.07 (0.04.to 0.10)	3 (2 to 4)	−0.03 (−0.06 to 0.00)	−1 (−3 to 0)	3.01 (2.97 to 3.05)	127 (126 to 129)	0.40 (0.38 to 0.42)	17 (16 to 18)
	**Influenza/pneumonia**	0.62 (0.14 to 1.11)	25 (6 to 45)	0.06 (−0.42 to 0.53)	2 (−17 to 22)	3.25 (2.66 to 3.84)	137 (112 to 162)	−0.05 (−0.50 to 0.40)	−2 (−21 to 17)
	**All respiratory**	1.36 (0.78 to 1.95)	56 (32 to 79)	−0.020 (−0.59 to 0.55)	−1 (−24 to 23)	2.89 (2.18 to 3.60)	122 (92 to 152)	−0.13 (−0.67 to 0.40)	−6 (−29 to 17)
**≥65 years**	Influenza only*	0.42 (0.34 to 0.49)	4 (3 to 5)	0.19 (0.11 to 0.27)	2 (1 to 3)	2.79 (2.69 to 2.88)	28 (27 to 28)	0.30 (0.24 to 0.36)	3 (2 to 4)
	**Influenza/pneumonia**	6.89 (4.27 to 9.52)	65 (40 to 90)	2.47 (−0.08 to 5.02)	24 (−1 to 49)	−5.60 (−8.80 to −2.40)	−55 (−87 to −24)	−0.03 (−2.45 to 2.39)	0 (−25 to 25)
	**All respiratory**	9.48 (6.15 to 12.81)	89 (58 to 121)	4.20 (0.96 to 7.43)	41 (9 to 72)	−7.78 (−11.84 to −3.72)	−77 (−117 to −37)	−0.11 (−3.18 to 2.96)	−1 (−33 to 30)

*Because of small numbers of influenza diagnoses outside of influenza seasons, predicted values were based on a constant mean value outside of influenza seasons. All other predicted values were based on the seasonally oscillating predicted values from the time series model.

The 2007 and 2009 influenza epidemics affected very different age groups. In 2009, the weekly rate of respiratory ICU admission in those aged 17 to 64 years peaked at 1.7 per 100,000 people in the week ending July 11 (Figure 3). In this age group, the total estimated excess rate of respiratory intensive care admissions was over twice that of 2007 (Table 1). By contrast, older people (65 years and older) were most affected in 2007, with a peak weekly rate of 6.7 per 100,000 people in the week ending July 21 (Figure 3). In 2009, the estimated impact of influenza on respiratory intensive care admissions in older people was negative, indicating that the observed intensive care admission rate in that age group was lower than the estimated seasonal baseline (Table 1).

**Figure 4:**
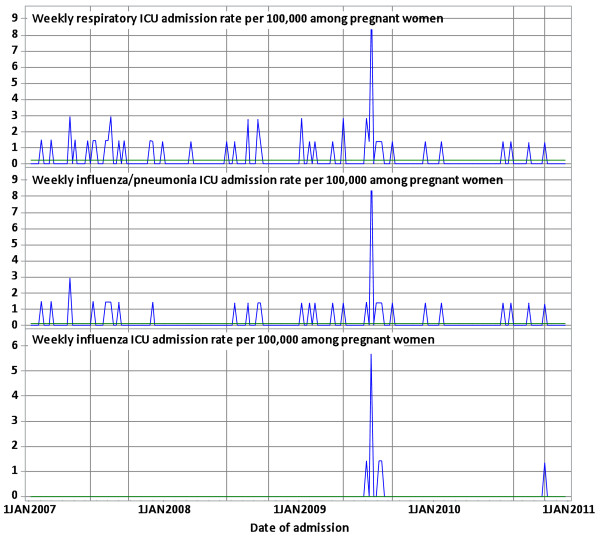
**Rates of intensive care admission in pregnant women.** Weekly observed (blue) and predicted (green) rates of intensive care admission per 100,000 among pregnant women of respiratory, influenza or pneumonia, or influenza illness, New South Wales, Australia, 2007 to 2010. Respiratory diagnoses include a primary diagnosis of any respiratory illness or a primary or other diagnosis of influenza or pneumonia. Each influenza period is marked with vertical reference lines.

**Figure 5:**
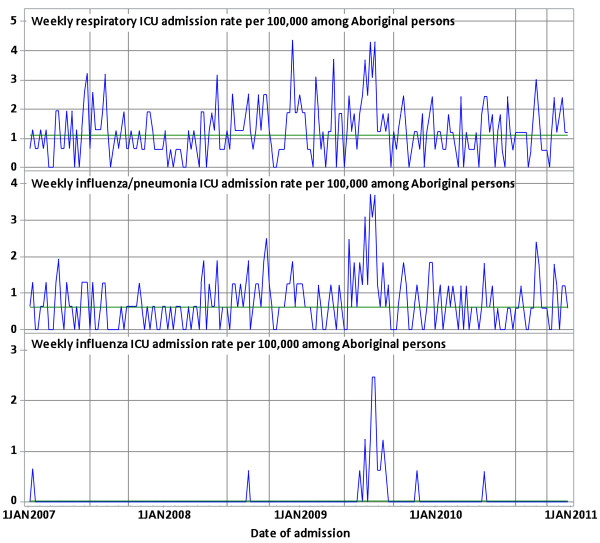
**Rates of intensive care admission in Aboriginal people.** Weekly observed (blue) and predicted (green) rates of intensive care admission per 100,000 among Aboriginal people of respiratory, influenza or pneumonia, or influenza illness, New South Wales, Australia, 2007 to 2010. Respiratory diagnoses include a primary diagnosis of any respiratory illness or a primary or other diagnosis of influenza or pneumonia. Each influenza period is marked with vertical reference lines.

Considering all respiratory intensive care admissions, pregnant/postpartum women were markedly more affected in 2009 (Table 2, Figure 4) with a rate of respiratory admission per 100,000 of 14.4 (95% CI 12.5 to 16.2), as were Aboriginal people with a rate of respiratory admission per 100,000 of 17.0 (95% CI 14.4 to 19.6) (Table 2, Figure 5). These compare to rates of 8.5 per 100,000 (95% CI 7.0 to 9.9) and 3.1 per 100,000 (95% CI 1.1 to 5.2) respectively for pregnant women and Aboriginal people in 2007. Both pregnant women and Aboriginal people have a younger age distribution than the general population, and when compared to the excess respiratory rate in 17 to 64 year olds, the excess rate in pregnant women was nearly 5 times larger, while the excess rate in Aboriginal people was nearly 6 times larger. This compares to differences of 6 times and 2 times, respectively, in 2007 (Table 2).

**Table 2:** Estimated differences in observed and predicted rates and counts of intensive care admissions, 2007–2010

		**Influenza season**							
		**2007 (15 weeks)**		**2008 (16 weeks)**		**2009 (19 weeks)**		**2010 (12 weeks)**	
		**Rate difference per 100,000 (95% CI)**	**Count difference (95% CI)**	**Rate difference per 100,000 (95% CI)**	**Count difference (95% CI)**	**Rate difference per 100,000 (95% CI)**	**Count difference (95% CI)**	**Rate difference per 100,000 (95% CI)**	**Count difference (95% CI)**
**Pregnant/ postpartum women**	Influenza only*	0	0	0	0	9.93	7	1.36	1
	**Influenza/pneumonia^**	5.27 (4.16 to 6.38)	4 (3 to 4)	3.41 (2.22 to 4.59)	2 (2 to 3)	13.00 (11.59 to 14.40)	9 (8 to 10)	1.09 (0.20 to 1.97)	1 (0 to 1)
	**All respiratory^**	8.48 (7.04 – 9.91)	6 (5 to 7)	4.95 (3.42 to 6.48)	4 (2 to 5)	14.36 (12.54 to 16.17)	10 (9 to11)	0.15 (−1.00 to 1.30)	0 (−1 to 1)
**Aboriginal**	**Influenza only^**	−0.19 (−0.41 to 0.02)	0 (−1 to 0)	0.42 (0.19 to 0.65)	1 (0 to 1)	10.86 (10.58 to 11.13)	17 (17 to 18)	−0.15 (−0.33 to 0.02)	0 (−1 to 0)
	**Influenza/pneumonia^**	−2.94 (−4.29 to −1.60)	−5 (−7 to −2)	7.59 (6.15 to 9.02)	12 (10 to 14)	17.15 (15.45 to 18.85)	27 (25 to 30)	2.15 (1.07 to 3.22)	4 (2 to 5)
	**All respiratory^**	3.12 (1.05 to 5.19)	5 (2 to 8)	7.85 (5.64 to 10.06)	12 (9 to 16)	17.01 (14.39 to 19.64)	27 (23 to 31)	0.44 (−1.22 to 2.09)	1 (−2 to 3)

*For influenza in pregnant/postpartum women, the rate outside of influenza seasons was zero and therefore no confidence intervals could be calculated.

^Because of small numbers of influenza diagnoses outside of influenza seasons, predicted values were based on a constant mean value outside of influenza seasons.

**Figure 6:**
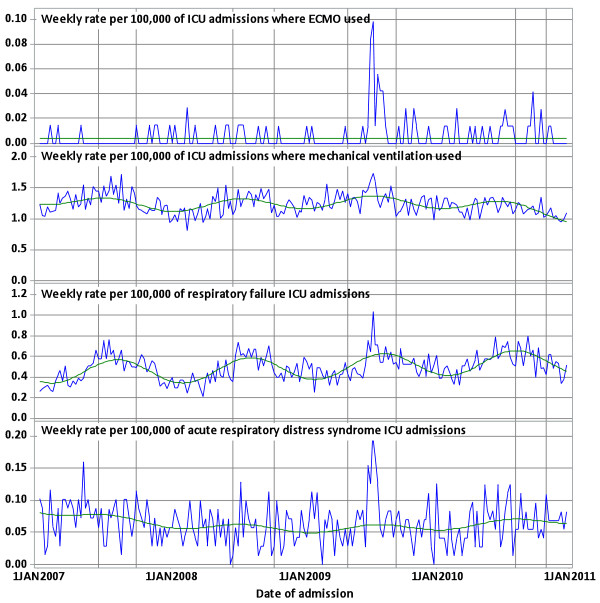
**Rates of intensive care admission involving ARDS, respiratory failure, mechanical ventilation, or ECMO.** Weekly observed (blue) and predicted (green) rates per 100,000 persons of intensive care admissions for acute respiratory distress syndrome, respiratory failure, where mechanical ventilation was used, and where extracorporeal membrane ventilation was used, New South Wales, Australia, 2007 to 2010. Each influenza period is marked with vertical reference lines.

In terms of severity, while respiratory failure and mechanical ventilation were more common in 2007, 2009 saw the greatest number of diagnoses of ARDS and the greatest number of admissions where the patient required ECMO, with an excess of 34 diagnoses (95% CI 21 to 46) and 21 admissions (95% CI 20 to 23) respectively (Table 3, Figure 6). No other year experienced an excess of ARDS diagnoses, while the next highest excess number of admissions where the patient required ECMO was observed in 2010, with an excess of 3 admissions (95% CI 2 to 4). Overall, in 2009 the median time spent in ICU in patients with respiratory illness and influenza/pneumonia were 77 hours and 96 hours respectively, which are similar to other years (Table 4). People under 65 years with influenza/pneumonia tended to have a longer stay in intensive care on average in 2009, while people 65 years and older had shorter stays (Table 4). In 2009 the percentage of respiratory patients who died while in intensive care was the lowest of all 4 years (16%) (Table 5).

**Table 3:** Estimated differences in observed and predicted rates and counts of intensive care admissions, 2007–2010

	**Influenza season**							
	**2007 (15 weeks)**		**2008 (16 weeks)**		**2009 (19 weeks)**		**2010 (12 weeks)**	
	**Rate difference per 100,000 (95% CI)**	**Count difference (95% CI)**	**Rate difference per 100,000 (95% CI)**	**Count difference (95% CI)**	**Rate difference per 100,000 (95% CI)**	**Count difference (95% CI)**	**Rate difference per 100,000 (95% CI)**	**Count difference (95% CI)**
**ARDS**	−0.05 (−0.19 to 0.10)	−3 (−13 to 7)	−0.08 (−0.22 to 0.068)	−5 (−15 to 5)	0.47 (0.29 to 0.65)	34 (21 to 46)	−0.02 (−0.15 to 0.12)	−1 (−11 to 9)
**Respiratory failure**	0.60 (0.068 to 0.94)	35 (5 to 65)	0.55 (0.12 to 0.96)	38 (8 to 68)	−0.21 (−0.74 to 0.32)	−15 (−53 to 23)	−0.27 (−0.68 to 0.13)	−20 (−49 to 9)
**Mechanical ventilation**	1.45 (0.75 to 2.14)	100 (52 to 148)	0.45 (−0.22 to 1.13)	32 (−15 to 79)	0.27 (−0.57 to 1.11)	19 (−41 to 79)	−0.069 (−0.71 to 0.57)	−5 (−51 to 41)
ECMO*	−0.05 (−0.06 to −0.03)	−3 (−4 to −2)	0.01 (−0.01 to 0.03)	0 (−1 to 2)	0.30 (0.28 to 0.32)	21 (20 to 23)	0.05 (0.03 to 0.06)	3 (2 to 4)

ARDS=Acute respiratory distress syndrome; ECMO=Extracorporeal membrane oxygenation.

*Because of small numbers of influenza diagnoses outside of influenza seasons, predicted values were based on a constant mean value outside of the influenza seasons. All other predicted values were based on the seasonally oscillating predicted values from the time series model.

**Table 4:** Median hours spent in intensive care during influenza periods, 2007–2010

		**Influenza season**							
		**2007 (15 weeks)**		**2008 (16 weeks)**		**2009 (19 weeks)**		**2010 (12 weeks)**	
		**n**	**Median (IQR)**	**n**	**Median (IQR)**	**n**	**Median (IQR)**	**n**	**Median (IQR)**
**All ages**	**Influenza/pneumonia**	774	89 (148)	749	95 (156)	1006	96 (175)	521	94 (178)
	**All respiratory**	1268	74.5 (123.5)	1215	82 (136)	1609	77 (125)	835	75 (135)
**<17 years**	**Influenza/pneumonia**	40	53 (165.5)	19	43 (150)	68	141.5 (223)	26	60.5 (74)
	**All respiratory**	122	42 (107)	67	43 (74)	201	65 (114)	94	52 (73)
**17 to 64 years**	**Influenza/pneumonia**	267	96 (198)	258	105 (209)	460	121 (216)	199	113 (196)
	**All respiratory**	452	74 (148)	422	85.5 (157)	656	90.5 (171.5)	307	92 (154)
**≥65 years**	**Influenza/pneumonia**	467	86 (124)	472	92 (143.5)	478	78 (118)	236	89 (177)
	**All respiratory**	694	82 (115)	726	83 (122)	752	70 (103.5)	434	74.5 (134)

**Table 5:** Count and proportion of intensive care admissions where the patient died, 2007–2010

		**Influenza season**			
		**2007 (15 weeks)**	**2008 (16 weeks)**	**2009 (19 weeks)**	**2010 (12 weeks)**
		**n (%)**	**n (%)**	**n (%)**	**n (%)**
**All ages**	**Influenza/pneumonia**	185 (23.9)	190 (25.4)	187 (18.6)	107 (20.5)
	**All respiratory**	247 (19.5)	250 (20.6)	250 (15.5)	151 (18.1)
**<17 years**	**Influenza/pneumonia**	0 (0)	1 (5.3)	2 (2.9)	3 (11.5)
	**All respiratory**	0 (0)	2 (3.0)	3 (1.5)	6 (6.4)
**17 to 64 years**	**Influenza/pneumonia**	44 (16.5)	49 (19.0)	58 (12.6)	23 (11.6)
	**All respiratory**	58 (12.8)	58 (13.7)	72 (11.0)	31 (10.1)
**≥65 years**	**Influenza/pneumonia**	141 (30.2)	140 (29.7)	127 (26.6)	81 (27.4)
	**All respiratory**	189 (19.5)	190 (26.2)	175 (23.3)	114 (26.2)

## Discussion

In NSW, when the entire influenza period is considered for each year and the age distribution of influenza-related illness is ignored, the population rate of influenza-associated respiratory admissions to intensive care was greater during seasonal influenza in 2007 than in pandemic influenza in 2009, despite the well-documented severe pandemic influenza-related illness in 2009. The perception of 2009 as a more severe influenza year than 2007 could be due to a number of factors, including the rapid progression of the epidemic of influenza A(H1N1)pdm09 in Australia, the intense media attention and uncharacteristic reporting of counts of confirmed cases and deaths, and the apparent increase in incidence of clearly influenza-related severe acute respiratory distress in younger adults compared with seasonal influenza epidemics. The rapid increase of the 2009 epidemic was followed by an equally rapid decline, thus tempering the final impact. In seasonal influenza years such as 2007, in which older persons were at greater risk of poor outcomes of influenza infection, the role of influenza may be less clear. A large proportion of patients hospitalised with seasonal influenza have symptoms uncharacteristic of a classic influenza syndrome [20,21].

In 2009, there is some evidence that patients who required intensive care due to influenza-related illness were more severely ill, with a large number of patients diagnosed with ARDS or who received ECMO. Further, while younger patients had a longer median stay in intensive care in 2009 than in other years, these figures should be interpreted with caution as only a small proportion of admissions would be truly due to influenza, and variation in other, non-influenza causes may have explained the differences.

In Australia, 2007 was considered a moderate to severe influenza season, which was attributed to antigenic drift in both the H1N1 and H3N2 influenza strains which may have compromised the effectiveness of that year's Southern Hemisphere influenza vaccine [22]. Even so, the 2009 period was highly unusual in that increases in influenza-associated intensive care admissions were observed only in people under 65 years. Older people were relatively protected, compared to normal seasonal influenza epidemic periods. Studies both in Australia and internationally have found that older populations tended to have a high prevalence of pre-existing cross-reacting influenza A(H1N1)pdm09 antibodies, while few younger people had such protection [23-25]. Hence, the all ages excess rate in 2009 was small when compared to 2007, as the increased rate in younger people was offset by the unusually low rate in older people. This was not the case in 2007, where rates were higher than expected in all age groups. A study from Denmark examining all influenza-associated hospital admissions also did not find an increase during the first epidemic of influenza A(H1N1)pdm09 virus compared with seasonal influenza, except in people younger than 65 years [26]. Our findings are consistent with those of a study in a US hospital that found that patients at greatest risk of complications from seasonal influenza tend to be older, with a mean age of 75 years [27]. The 2010 epidemic, the first season following the arrival of pandemic influenza continued to be dominated by the pandemic strain. This season was characterised by low influenza activity and very low influenza-related ICU use.

For 2009, we estimated the rate of influenza-related respiratory ICU admission among Aboriginal people to be more than 6 times the highest rate in any single age group of the population overall. In 2009, it was recognised that Indigenous people around the world experienced a greater risk of infection with the pandemic influenza virus and more severe outcomes [28-30] although a Canadian study found no increased risk of intensive care admission or death among the Canadian Aboriginal populations [31]. A more recent study of Australian Aboriginal and Torres Strait Islander people found that a younger age distribution and a higher prevalence of underlying chronic conditions are a possible explanation for the apparent increased risk of influenza-related hospitalisation [32]. Improvements in the recording of Aboriginal status in hospitalisation data in NSW could affect the comparability of rates over time.

The younger risk profile of 2009 pandemic influenza may also at least partly explain the higher rate of influenza-related ICU admission among pregnant women compared with the overall population. However, in all years the risk of admission was greater in pregnant women than in the general population, indicating that seasonal influenza can also pose a strong risk to pregnant women.

The use of administrative data in this study has both strengths and limitations. The main strength of this study is that it is population based which allows complete capture of all intensive care admissions in NSW residents. However, there is certainly some difficulty in accurately and consistently identifying admissions associated with influenza infection over time. There are many non-influenza causes of respiratory illness, both infectious and non-infectious. Other viruses, such as respiratory syncytial virus, may have also been circulating during the influenza periods. We controlled for these non-influenza factors using the Serfling method. However, a limitation of the Serfling method is that it generates a rigid expectation of non-influenza seasonal illness. While the sinusoidal model follows the background seasonal pattern, some variation in non-influenza causes of illness from year-to-year could lead to some over- or underestimation in some years. Other time series methods for estimating influenza-attributable health outcomes are available, such as a combined Serfling-Poisson model that incorporates virology data, but these have been shown to produce broadly similar results [33,34]. The lack of a gold standard for assessing the accuracy of these estimates remains a barrier for objectively comparing methods.

Management of ECMO services was coincidentally altered in NSW in 2009 prior to the emergence of the pandemic influenza strain. The aim was to increase the mobility of ECMO services to allow wider geographic application. This may also have contributed to the apparent increase in use of ECMO associated with the 2009 epidemic. In addition, this increased use may reflect the younger age distribution in 2009, given that clinicians may be more likely to use extreme measures on younger patients.

Since there is no generally accepted definition of an influenza season, we used a threshold of 3% of respiratory samples testing positive for influenza to define the seasons. While different thresholds can give slightly different results [35], the 2009 season as defined by our method is consistent with other estimates [3,36].

This study highlights the challenge of identifying influenza-related illness. Had we only included ICU admissions where influenza was recorded as a diagnosis, we would have concluded that there was a much greater use of influenza-related intensive care during 2009 than 2007. Prior to 2009 specific testing for influenza in ICUs was uncommon, and during 2009 we believe that pandemic awareness amongst clinicians promoted testing for influenza, and increased the likelihood that influenza was recorded as part of the diagnosis, as evidenced by Figure 1. As well, a shift between 2007 and 2009 from the use of immunofluorescence for the detection of influenza to the more sensitive nucleic acid testing may have contributed to the increased rate of influenza diagnosis in 2009.

Including all respiratory admissions is a more sensitive method of estimating the impact of influenza. The influence of influenza during 2007 may have been more evident in the broader respiratory diagnosis group due to the older age of those most at risk of influenza complications in 2007. Older people have more complex and chronic illnesses which could mask the recognition of influenza's role in the illness and lead to more frequent diagnoses of alternative conditions such as pneumonia and respiratory failure/end-stage respiratory disease that could be secondary respiratory complications of primary influenza infection [11,37].

## Conclusions

While influenza was diagnosed more frequently and peak use of intensive care was higher in 2009, a broader assessment of respiratory admissions to intensive care indicates that 2007 seasonal influenza resulted in a higher rate of excess admissions to ICU than 2009 pandemic influenza. Thus, the role of seasonal influenza epidemics on intensive care use may have previously been under-recognised. In 2009, relatively high use of intensive care among young to middle-aged adults was offset by low use among adults aged over 65 years. Compared with seasonal influenza, the pandemic virus in 2009 was associated with some increase in markers of disease severity, but no overall increase in hospital mortality.

Pandemic influenza in 2009 had a far greater impact on Aboriginal people and pregnant women compared both with the overall population in 2009 and with seasonal influenza in other years. Future pandemic planning should place a priority on prevention of serious illness in these groups.

## Key messages

Excess intensive care admission rates for respiratory illness associated with pandemic influenza in 2009 were lower than that during the influenza season of 2007. Thus, the role of seasonal influenza on use of intensive care services may have previously been under-recognised.

Elderly persons accounted for much of the influenza-associated intensive care use from seasonal influenza, while young to middle-aged adults accounted for the majority of the pandemic influenza-related use.

Aboriginal people and pregnant women were substantially over-represented among intensive care admissions during pandemic influenza in 2009. A priority in influenza pandemic planning should be prevention of serious illness in these groups.

## Abbreviations

CI: Confidence interval; ICU: Intensive care unit; NSW: New South Wales; APDC: Admitted Patients Data Collection; ICD-10-AM: International Classification of Diseases, 10^th^ Revision. Australian Modification; ACHI: Australian Classification of Health Interventions; ECMO: Extracorporeal membrane oxygenation; ARDS: Acute respiratory distress syndrome.

## Competing interests

The authors declare that they have no competing interests.

## Authors’ contributions

DM, ST and RG conceived the study. AS, DM, ST and MC designed the study. AS carried out the data analysis. AS and DM drafted the manuscript. ST, RG, MC, and JW contributed to the interpretation of the results and revised and critically reviewed the manuscript. All authors have read and approved the final manuscript.

## Pre-publication history

The pre-publication history for this paper can be accessed here:

http://www.biomedcentral.com/1471-2458/12/869/prepub
